# Simple diagnosis of cancer by detecting CEA and CYFRA 21-1 in saliva using electronic sensors

**DOI:** 10.1038/s41598-022-19593-8

**Published:** 2022-09-12

**Authors:** Sowmya Joshi, Shashidhar Kallappa, Pranjal Kumar, Sudhanshu Shukla, Ruma Ghosh

**Affiliations:** 1 Department of Electrical Engineering, Indian Institute of Technology Dharwad, Dharwad, 580011 Karnataka India; 2 Department of Surgical Oncology, Karnataka Institute of Medical Sciences, Hubli, 580029 Karnataka India; 3 Department of Biosciences and Bioengineering, Indian Institute of Technology Dharwad, Dharwad, 580011 Karnataka India

**Keywords:** Biomarkers, Health care, Oncology, Graphene, Techniques and instrumentation

## Abstract

One way of early diagnosis of cancer is by detecting the biomarkers that get introduced into easily accessible body fluids. We report the development of portable and rapid electronic biosensors for quantitative detection of two secretive cancer biomarkers–Carcinoembryonic antigen (CEA) and Cytokeratin fragment 19 (CYFRA 21-1). The reduced graphene oxide (rGO)/ melamine (MEL)/antibodies/ bovine serum albumin (BSA) based devices were tested for 1 pg/mL to 800 ng/mL of CEA and CYFRA 21-1. The responses of the sensors ranged from 7.14 to 59.1% and from 6.18 to 64% for 1 pg/mL to 800 ng/mL CEA and CYFRA 21-1 respectively. A read-out circuit was assembled to develop a portable prototype which was used to assess the concentrations of the two antigens present in saliva samples of 14 subjects. The prototype could accurately discriminate between 9 oral squamous cell carcinoma patients and 5 healthy controls.

## Introduction

Cancer is one of the leading causes of death worldwide^1–3^. Around 9.5 million lives were claimed due to cancer in the year 2018 and this burden is expected to increase to around 16 million by the year 2040^4^. The primary cause for such high fatality associated with the disease is that cancers of almost all types are diagnosed at advanced stages. This happens due to the lack of availability of easy and rapid screening technologies for early cancer diagnosis^5^. Usually, patients in the early stages of cancer, experience symptoms that overlap greatly with the symptoms of minor illnesses, nominal infections, ulcers, cold, and cough^6^. As a result, patients are hesitant to get themselves checked at such stage because present diagnostic approaches like tomographic scans^7^, X-ray imaging^8^, sputum cytology^9^, biopsy of tissues^10^, etc. are expensive, lab-intensive, and sometimes invasive. This prompts the necessity to develop inexpensive, highly sensitive, and portable biosensors that need a small volume of easily collectable patients’ samples (saliva, sputum, urine, etc.), require very less or no additional chemicals, and enable quick diagnosis^11^. Such biosensors are expected to be of great clinical interest and subsequently, play a pivotal role in point-of-care (POC) testing applications^12^. One of the efficient ways in which this could be achieved is by developing biosensors for the secretive biomarkers of cancer which get introduced into easily accessible bodily fluids (saliva, sputum, blood, urine, etc.) at an early stage of the cancer^13^.

Carcinoembryonic antigen (CEA) and cytokeratin fragment-19 (CYFRA 21-1) are two such biomarkers of multiple cancers including lung cancer, oral cancer, colorectal cancer, etc.^14^. The biggest merit of these antigens is that they can also be found in the saliva of the patients^15^. CEA and CYFRA 21-1 are found in the saliva of healthy people too but their concentrations were found to be very low (0–3 ng/ml)^16^. The concentrations of these antigens were observed to increase beyond 5 ng/mL in the saliva samples of the patients suffering from cancer. Thus, detecting CEA and CYFRA 21-1 simply and easily might be a key not only to diagnosing the set of terminal diseases early but also in monitoring the tumour progressions and evaluating the different types of treatment of cancer^17,18^.

Several methods have been explored and extensively investigated for detecting CEA and CYFRA 21-1. One such method is enzyme-linked immunosorbent assay (ELISA)^19^ in which antibodies or antigens are labelled with enzymes, the antigens are specifically immobilised in liquid phase, and the interaction is detected through coloration caused by an enzymatic reaction between the assay and the chromogenic substrate. Although ELISA is a commercially available technique for detecting biomarkers, it is labor and lab-intensive and thus unsuitable for developing POC devices for lung cancer. It also has low sensitivity (~ 0.1 ng/mL), which frequently leads to incorrect results^20^. Other methods include electrochemical sensors, electrochemiluminescence (ECL), photoelectrochemical immunosensors, and surface-enhanced Raman scattering (SERS) based sensors^19–25^. These techniques use chemiluminescence, photochemistry, fluorescence, or changes in optical or electrical (current/voltage) properties of the device to capture the antibody-antigen interaction. These approaches except electrochemical sensors, however, need large apparatus, skilled expertise in handling the facility and capturing the interaction, and are costly^26^. Electrochemical sensors offer the advantages of relatively simpler operation and are portable but they require electrolytes (which are mostly liquid, suspensions, or colloids) for their operations thus, can offer limited lifetimes. Also, the scope of miniaturization and making the devices compact is restricted^24^. As a result, none of these approaches are viable candidates for POC devices of cancer.

Antigens and antibodies are charged particles and their interactions lead to the change in cumulative charge densities^27^. So, one of the simple techniques of detecting the antigens is by capturing the electrical signals that are produced due to the antigen–antibody interactions^28^. The first step towards this is the immobilization of the antibodies on a base substrate. The immobilization requires surface modification of the base layer using suitable agents. In search of such functional immobilizing materials for biosensors, a wide variety of materials that can interact with the antibodies via covalent or non-covalent bonding have been explored^27–29^. However, all of the above-mentioned materials suffer from one or more limitations, including lack of structural tunability, expensive, particle agglomeration, etc. To achieve complete functioning, the antibodies' conformations must not be changed, and their active sites must not be obscured during immobilisation. Each antibody has two fragment antigen binding (F_ab_) regions and one fragment crystallisable (F_c_) region^32^. These fragments contain amine and carboxyl groups throughout an antibody's structure and, due to their polar nature, are abundant near the antibody's surface. As a result, presence of active functional groups like amine, thiol, and aldehyde groups formed by carbohydrate oxidation in the F_c_ on the surface of the immobilizing layer may aid in the anchoring the antibody on its surface^32,33^. In addition, the antibody immobilizing layer should be such that it can anchors the antibodies without changing the specificity and immune activities of the same. One such attempt is to exploit the covalent immobilization technique using amine coupling method. This method is known as an effective way for immobilising proteins or small molecules by using (1-ethyl-3-(3-dimethylaminopropyl)carbodiimide hydrochloride) (EDC)/N-hydroxysuccinimide (NHS)^34,35^. A simple alternative of EDC/NHS is using melamine (MEL) for amine coupling method. Each melamine monomer has three unshared pairs of electrons accessible as hydrogen bond acceptors due to the *sp*^*2*^ hybridised nitrogen atoms of its triazine ring. Unsubstituted MEL also contains three exocyclic primary amines, each of which has the ability to give two hydrogen bond donors^36^. This enables a stable H-bonding of the antibodies to the surface.

Next important constituent of biosensors that are designed to capture the electrical signals coming out of the antigen–antibody interactions is the base transport layer. The antibody immobilization matrices are usually non-conducting or semiconducting material which cannot be used as is to faithfully transport the change in total charge densities to the measuring instruments^37^. Two-dimensional nanomaterials are expected to play a pivotal role in such electronic transportation. Reduced graphene oxide (rGO) is a carbon nanomaterial which is a graphene derivative with hexagonal rings of *sp*^*2*^ hybridised C-atoms. In addition, multiple functional groups are attached to the edges and basal planes of rGO^38^. It has exceptional mechanical and electronic properties which include high carrier mobilities. This is an extremely important property for developing biosensors with an intention to capture electronic signals generated by the biomarkers^39^.

This study reports development of a simple, label-free electronic sensor which can be used to detect wide range of concentrations of CEA and CYFRA 21-1 biomarkers. The sensors use rGO as their base transport layer and nitrogen rich MEL as the antibody immobilizing layer. The role of amine groups present in the MEL in anchoring the antibodies has been established by comparing the response with triazine which has less amine groups available in it than MEL. The antigens were introduced to the sensors simply by dropping them on the rGO/MEL/antibody/BSA devices at room temperature under ambient conditions. The current flowing through the devices was measured after coating rGO by applying a DC voltage of 3.3 V. The change in the current flowing through the sensors after binding the antigens was taken as the sensors’ signals. The devices were tested for eight different concentrations of CEA and CYFRA 21-1. Despite some similarities between the developed device and the electrochemical sensors, there are distinct differences which are reported in one of earlier researches^40^. As a primary step to develop a prototype of the electronic biosensor and to make the device portable, a simple readout circuit was developed using an auto range ohmmeter circuit. The developed prototype was tested with human saliva samples and the concentrations of CEA and CYFRA 21-1 present in all the real-life samples were ascertained. The predicted concentrations of CEA were also cross validated with a commercial ELISA kit of human CEA. The obtained satisfactory results of the sensor device indicated a potential practical application in clinical diagnostics. The results have been presented and discussed in detail in subsequent sections.

## Results

### Material characterizations

**Figure 1:**
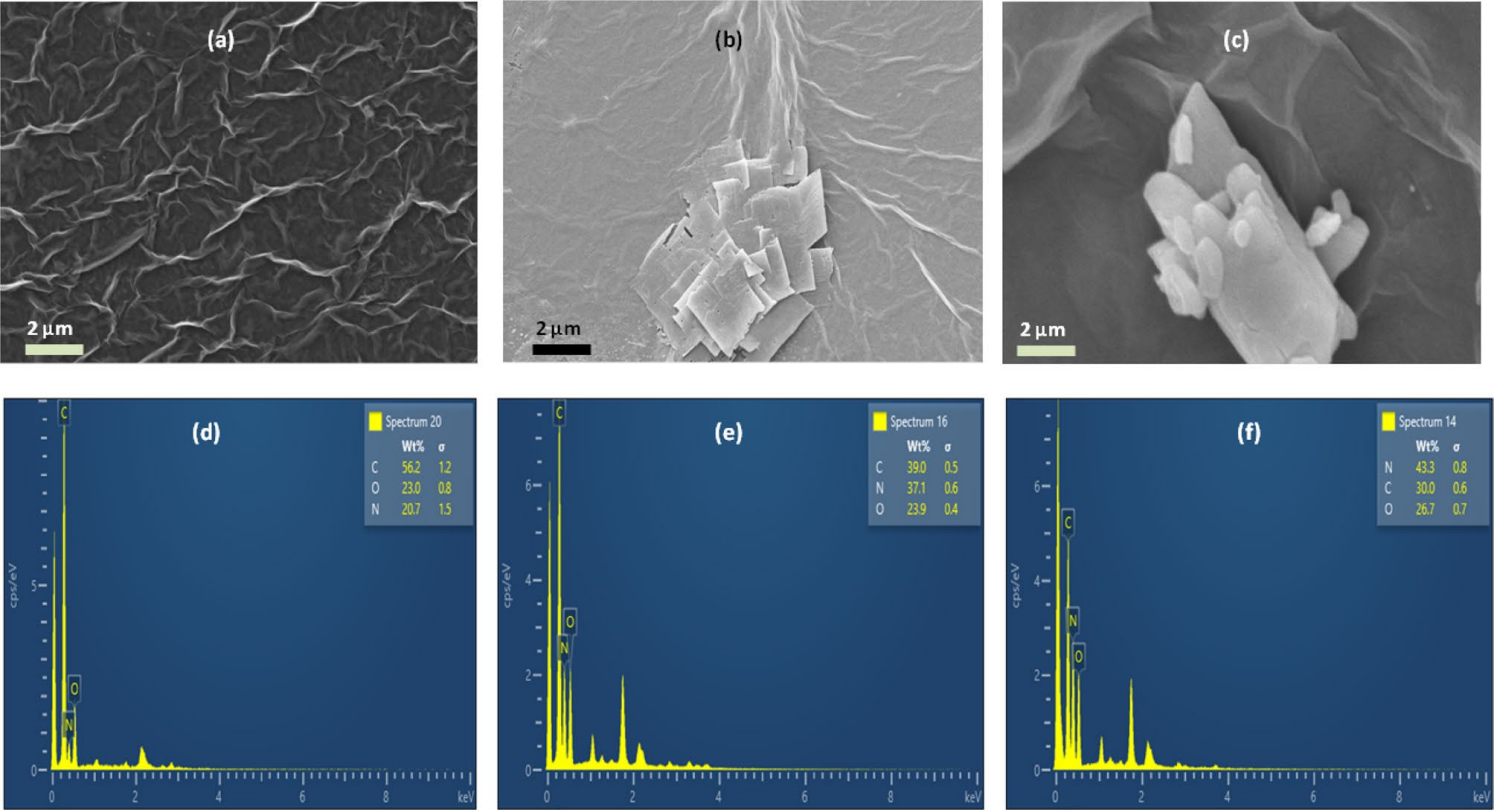
(**a**–**c**) FESEM images. (**a**) rGO, (**b**) rGO/MEL, (**c**) rGO/Triazine, (**d**–**f**) EDS of (**d**) rGO/Triazine, (**e**) rGO/MEL, (**f**) rGO/MEL/antibody.

The first step of the rGO synthesis was exfoliating the bulk graphite powder to graphene (GO) using Hummer’s method^40^. The thickness of GO samples was ascertained using atomic force microscopy (AFM) and the average thickness was found to be a few nm (Fig. S1). This confirmed that the top-down approach of exfoliating bulk graphite powder was successful and few layered GO sheets could be effectively isolated. Next, it was critical to investigate the surface morphologies of the biosensor's various constituents. Figure 1a–c show field emission scanning electron microscopy (FESEM) images of rGO, rGO/MEL, and rGO/Triazine layers. rGO is a two-dimensional carbon nanomaterial with a crumpled and wrinkled sheet-like morphology when deposited on any substrate (Fig. 1a). Figure 1b shows the coexisting crumpled thin rGO sheets and square plates of MEL whereas Fig. 1c shows the rGO/Triazine sample in which a thick triazine flake and the thin rGO sheets in the background can be clearly seen. MEL has 1,3,5-triamino-2,4,6 triazine structure. Due to the presence of additional amine groups, the number of N-atoms present in MEL is expected to be more than that present in triazine. Energy dispersive spectroscopy (EDS) was employed to ensure the elemental composition of the different layers present in the biosensor devices. The EDS spectrum of rGO/triazine (Fig. 1d) sample exhibited presence of C, O and N. The C and O atoms belonged to the rGO layer and N comes from triazine. The wt% of N in rGO/MEL sample was found to be more than that present in rGO/triazine sample (Fig. 1e). As antibodies are rich in amino acids, the wt% of N atoms were observed to increase further in the EDS spectrum of rGO/MEL/antibody sample (Fig. 1f). All three layers contribute to C-atoms whereas MEL and CEA antibodies are responsible for the proportion of N-atoms present in the sample whereas O is contributed only by rGO.

The EDS spectra of all the samples also ensure the purity of the synthesized/coated materials.

### Biosensor characterizations

The currents flowing through the devices were found to decrease with introduction of the antigens on them. The response of the developed electronic sensors was calculated using Eq. (1)

1 $$Response\,\,(\% ) = \frac{{I_{0} - I_{antigen} }}{{I_{0} }} \times 100 = \frac{\Delta I}{{I_{0} }} \times 100$$

where *I*_antigen_ is the current measured across the sensor in presence of the antigen and *I*_0_ is the baseline current measured after rGO was coated on the device. The baseline range of the current that was maintained for carrying out all the experiments is 200 µA–1 mA.

**Figure 2:**
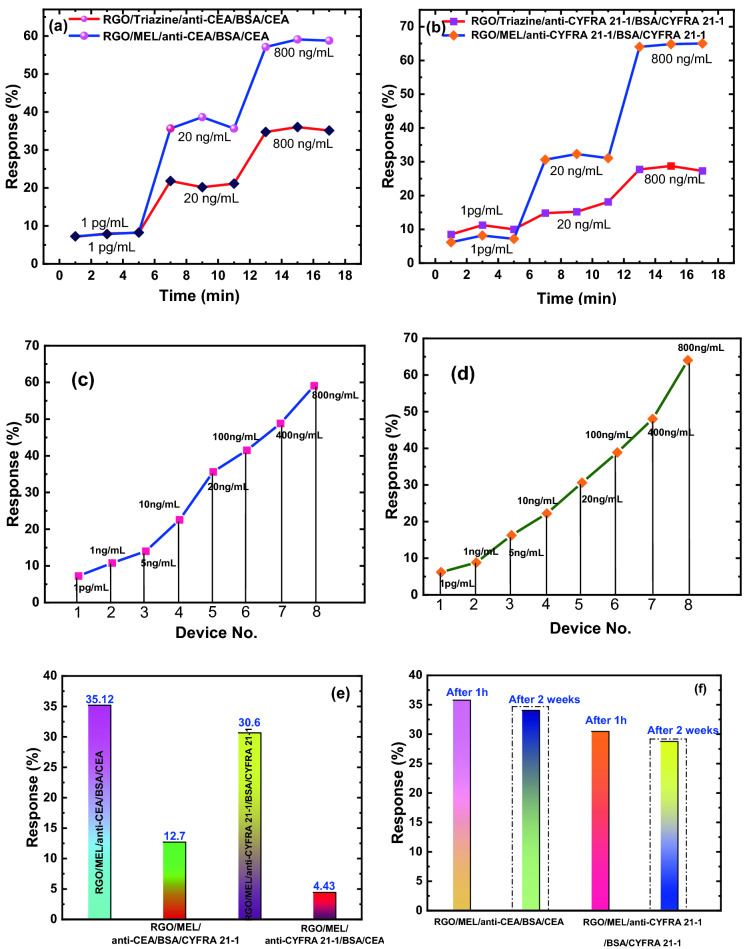
(**a**,**b**) Comparative response of rGO/MEL/antibody/BSA and rGO/Triazine/antibody/BSA for (**a**) CEA, (**b**) CYFRA 21-1, (**c**,**d**) response of (**c**) rGO/MEL/anti-CEA/BSA to 1 pg/mL to 800 ng/mL of CEA, (**d**) response of rGO/MEL/anti-CYFRA 21-1/BSA/ to 1 pg/mL to 800 ng/mL of CYFRA 21-1 at room temperature. (**e**) Cross-reactivity results, (**f**) stability test results.

In an attempt to understand the cardinal role of amine groups in the antibody binding and subsequently, in the biosensor performance, rGO/MEL/antibody/BSA and rGO/Triazine/antibody/BSA devices were fabricated and tested for three different concentrations of both the biomarkers. Figure 2a,b show the relative responses of three devices tested each for 1 pg/mL, 20 ng/mL and 800 ng/mL of CEA (Fig. 2a) and CYFRA 21-1 (Fig. 2b). For lower concentration (1 pg/mL) of the biomarkers, the performances of the triazine based devices are observed to be almost same or slightly better (in case of CYFRA 21-1) than that of MEL based devices. This is because N-atoms also have affinity towards the antibodies^41^. Triazine has N-atoms (due to the imine group) available within it and these N-atoms are perhaps sufficient to anchor antibodies that are enough in amount to bind the antigens present in low concentration samples. Hence, the effect of amine groups in addition to the N-atoms of imine groups, in the immobilizing layer from triazine to MEL was not noticeable. However, when the concentrations of the antigens increased, the presence of amine groups and subsequently, immobilization of higher number of antibodies on the sensor devices became significantly important. Hence, the response of the rGO/MEL/antibody/BSA devices were found to be almost double than those of the triazine based devices as is evident from Fig. 2a,b.

Literature reports that the concentrations of CEA and CYFRA 21-1 in the saliva of patients suffering from cancer ranged from 3.3 to 5 ng/ml respectively to hundreds of ng/ml^42^. These concentrations are lower in case of healthy subjects as mentioned in introduction section. Therefore, the rGO/MEL/antibody/BSA biosensors were tested for a wide range of concentrations of CEA and CYFRA21-1 (1 pg/mL to 800 ng/mL) at room temperature which covered the concentrations of these biomarkers found in case of cancer as well those for the healthy subjects. The responses of the biosensors were found to vary as a function of the concentrations of the two antigens. The response of the sensors was found to range from 6.1 to 59.1% for 1 pg/ml to 800 ng/ml CEA (Fig. 2c) and from 7.1 to 64.1% for 1 pg/ml to 800 ng/ml CYFRA 21-1 (Fig. 2d). The Limit of detection (LOD) of biosensors is another crucial parameter and is calculated through statistical methods. The details of the LOD calculation have been reported earlier^40^. The LOD of the sensors for CEA and CYFRA 21-1 was found to be 0.148 pg/mL and 0.04 pg/mL respectively (Fig. S2a–d).

During the preliminary experiments, it was discovered that the MEL concentration in the device had an impact on the device's performance. So, it was necessary to optimize the concentration of MEL present in the device. The optimum concentration of MEL in the biosensor was determined by fabricating numerous devices with varying MEL dispersion concentrations (1–8 mg/mL) and then assessing the performance of all the devices for one particular concentration of CEA and CYFRA 21-1, in our case, it was chosen to be 20 ng/ml. When compared to the rest of the concentrations of the MEL dispersion examined for CEA and CYFRA 21-1, the 4 mg/mL conc. of MEL had the highest response (35.64% and 30.63% respectively, Fig. S3). This might be because the lower concentration of MEL (1 mg/mL) does not provide enough anchoring sites to the antibodies, and when the concentration of MEL is increased beyond 4 mg/mL, the antibodies clump together on the sensor device and form agglomerates thereby reducing the sensor performances (Fig. S4). Hence, all the studies were carried out with 4 mg/mL MEL concentration.

A biosensor's selectivity is an essential aspect, in addition to its sensitivity. To assess the selectivity of the developed sensors, the rGO/MEL/anti-CEA/BSA device was tested with 20 ng/mL CYFRA 21-1 and the response was found to be 12.7% which is close to the response of the device to 1 pg/mL CEA. Similarly, rGO/MEL/anti-CYFRA 21-1/BSA device was tested with 20 ng/mL CEA and the response exhibited by the device for CEA was determined to be 4.43% as shown in Fig. 2e. This response is lesser than that shown towards 1 pg/mL CYFRA 21-1. This happened because we have used monoclonal antibodies for developing the sensors and the tested antigens were highly pure. Hence, the antigen–antibody bindings were specific so, the selectivity of the electronic sensors was excellent.

The intent of this work is to develop a portable device that can be used by the medical practitioners for easy and simple diagnosis of cancer. In such case, one of the pivotal performance metrics of the sensor is the stability i.e., the performance of the sensors should not deteriorate even if used after certain number of days. The developed biosensors were hence, assessed for stability by preparing the devices and keeping them at 4 °C for 2 weeks. These stored devices were then tested for 20 ng/mL of CEA and CYFRA 21-1. The sensors' responses were determined to be 33.95% and 28.7% for CEA and CYFRA 21-1 respectively (Fig. 2f), which were found close to the response of freshly developed biosensors.

Next, the performance of the sensors developed in this work was compared to that of other sensing methods that are commonly used to detect these protein biomarkers (Table 1). The developed sensors not only provided a simple method of detecting CEA and CYFRA 21-1, but also outperformed most of the available sensors of these biomarkers.

**Table 1:** Comparison between different biosensors reported for CEA and CYFRA 21-1 detection and the ones developed in this research.

Sl. no.	Type of sensor	Sensing material	Target Bio marker	Lowest measured concentration	References
1	ELISA	–	CEA, CYFRA-21	~ng/mL	^19^
2	Electrochemical immunosensor	Cubic CeO2 implanted reduced graphene oxide based biosensor	CYFRA 21-1	0.625 pg /mL	^43^
3	Electrochemical immunosensor	Polyhydroquinone-graphene composite	CYFRA-21	2.3 pg/mL	^44^
4	Electrochemical immunosensor	Amine-functionalized MoO3@RGO Nanohybrid-Based Biosensor	CEA	0.001 ng/mL	^45^
5	Electrochemical immunosensor	A sandwich-type electrochemical immunosensor using polythionine/AuNPs nanocomposites	CEA	0.3 ng/mL	^46^
6	This work	RGO/MEL/antibody/BSA/antigen	CEA, CYFRA-21	1 pg/mLfor both the antigens	

### Sensing mechanism

The novel electronic device developed in this study comprises of various layers, each of which plays distinct and critical role, as detailed below:

#### Role of MEL

**Figure 3:**
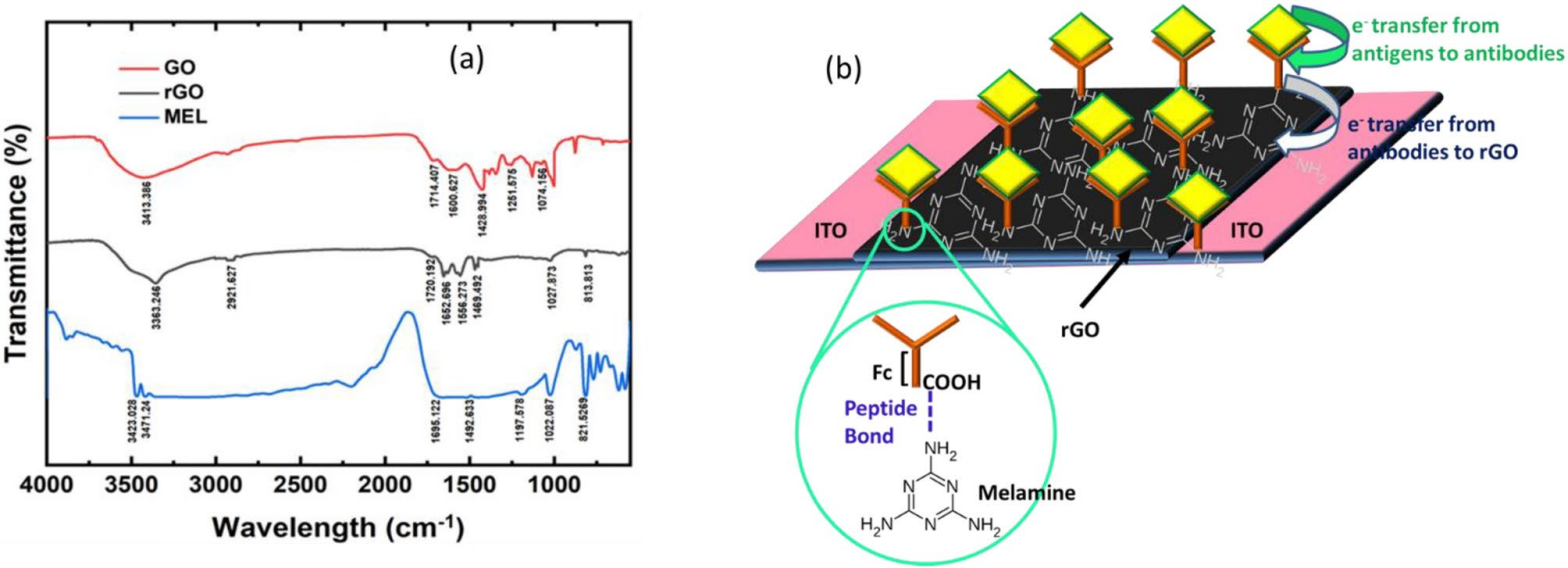
(**a**) FTIR spectra of GO, rGO and MEL. (**b**) Schematic depicting sensing mechanism.

Melamine and similar triazine derivatives can create self-assembling, high molecular weight complexes via structured intermolecular networks of H-bonding and/or peptide bonding (owing to the availability of free amines, carbonyl groups, and hydroxyls) with the carboxyl group present in the Fc domain of the antibodies. Figure 3a depicts the FTIR spectra of MEL which shows –NH_2_ vibrational modes (N–H stretching mode) at 3471.24 cm^−1^ and 3423.02 cm^−1^^47^. Similarly, the bands 1430–1700 cm^−1^ indicate the CN stretching and NH_2_ bending vibrations. MEL is well represented by the peak at 1638 cm^−1^, which is ascribed to the C=N stretching vibration. The bands in the lower wave number range (e.g., 821 cm^−1^, 1022 cm^−1^) show ring deformation modes^48^. Primary amine groups on MEL are targeted for covalent bonding with the peptide units and the N-glycans of Fc region of the antibodies ensures robust immobilization. MEL is however, insulating in nature and hence, if the thickness of the polymer increases on the rGO surface, then the information of the change in charge densities due to the antigen–antibody interactions do not get transferred to the base transport layer properly. It was evident from Fig. S3 which showed that as the amount of MEL in the biosensors increased, due to higher insulation, the net change in the electrical charge was prohibited to reach the base layer, rGO.

#### Role of rGO

As the antibody immobilizing agent was an insulating material, it was necessary to have a conductive layer beneath which could transport the change in the charge densities occurring during antigen–antibody interactions, faithfully to the measuring instruments. In addition, Fig. 3a shows the FTIR spectra of GO and rGO that confirms that GO has a large number of oxygenated groups^49,50^. The vibration peaks of GO at 2500–3500, 1724.04, 1251.5, and 1600.6 cm^−1^ are attributed to hydroxyl groups (OH), carbonyl groups (C=O), alkoxy (C–O–C), and carboxylic groups (COOH) respectively^51^. These groups are also expected to aid in antibody immobilization^52^. But GO like MEL, is electrically insulating and hence, it was necessary to reduce the GO, to regain the conductivity (Fig. S5). This step was a critical requirement for the base transport layer. While reducing the GO, the vibration peak at 1724 cm^−1^ corresponding to carbonyl groups vanishes in the rGO, and a new peak at 2921.6 cm^−1^ develops, indicating that the reduction of carbonyl groups to methylene group (–CH2–)^53^. It can also be seen that the amplitude of the signal at 1600.627 cm^−1^ is significantly lower in the rGO compared to the GO. The role of rGO in the device is as important as that of MEL, and without the rGO layer, the sensor's baseline (MEL/antibody) was found to be very low (~ nA), with no noticeable change in current after incubating the CEA/CYFRA 21-1.

Antibodies and antigens are oppositely charged species and their interactions to cause a reduction in the net charge of the clustered species. This information of the change in charge densities is transported to rGO layer as shown schematically in Fig. 3b. As a result, the net charge in the *p-*type rGO layer changes which is reflected as a decrease in current flowing through the rGO/MEL/antibody device.

### Readout circuit for portable biosensor

#### *Development of the algorithm for accurate quantification of CEA and CYFRA *21-1* concentration*

**Figure 4:**
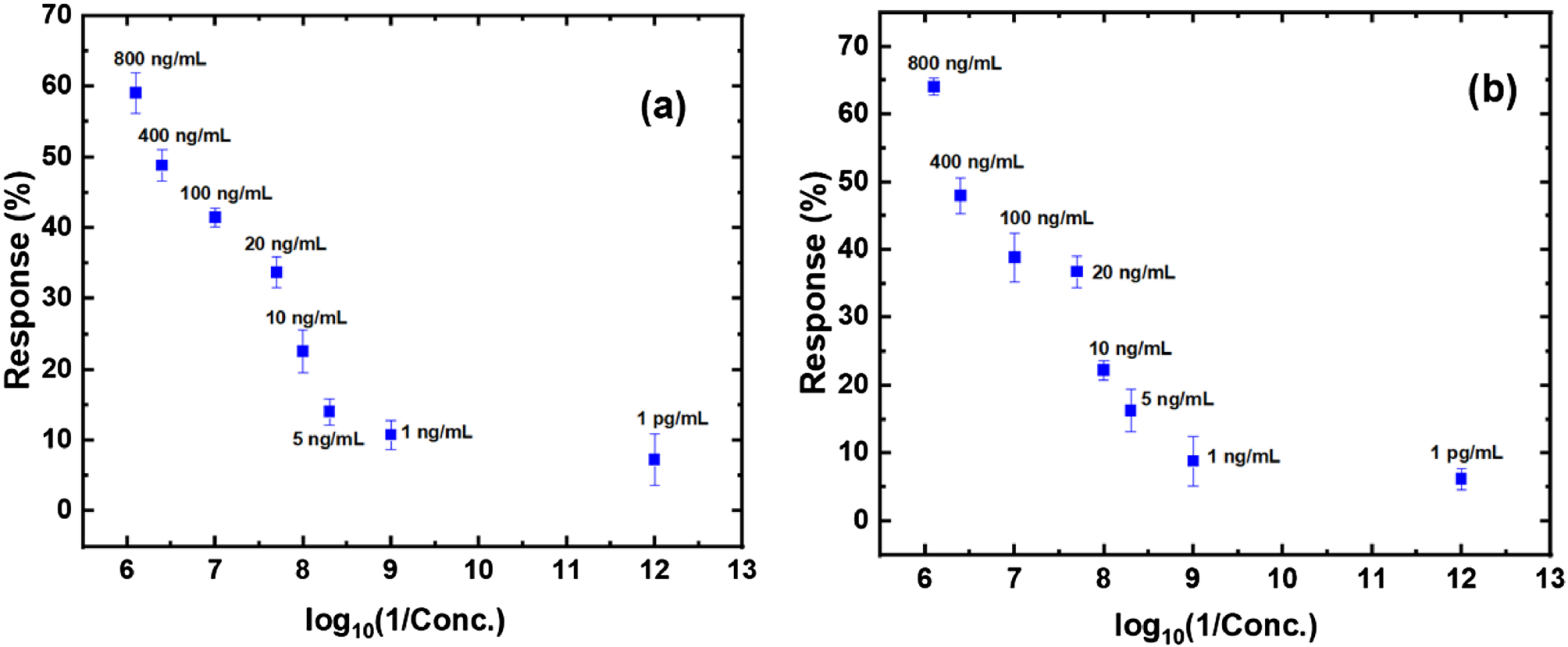
Variation in response of rGO/MEL/antibody/BSA devices for (**a**) CEA, (**b**) CYFRA 21-1.

To determine the concentrations of the antigens, numerous devices with different baseline currents were tested for all concentrations of CEA/CYFRA 21-1 and the variations in the response of the rGO/MEL/antibody/BSA devices was assessed. The differences in sensor response for different concentrations of CEA and CYFRA 21-1 are shown in Fig. 4a,b.

A total of 80 (5 devices for each concentration of CEA and CYFRA 21-1) devices were fabricated and evaluated for each concentration of the two antigens. Even though the baseline current (I_o_) was seen to vary owing to different factors including the number of layers coated, the sensors' responses were observed to be independent of the baseline currents of the devices. This is because of the formula that was used to calculate the response which effectively nullified the variations on the baseline current and considered only the relative change in the current.

**Figure 5:**
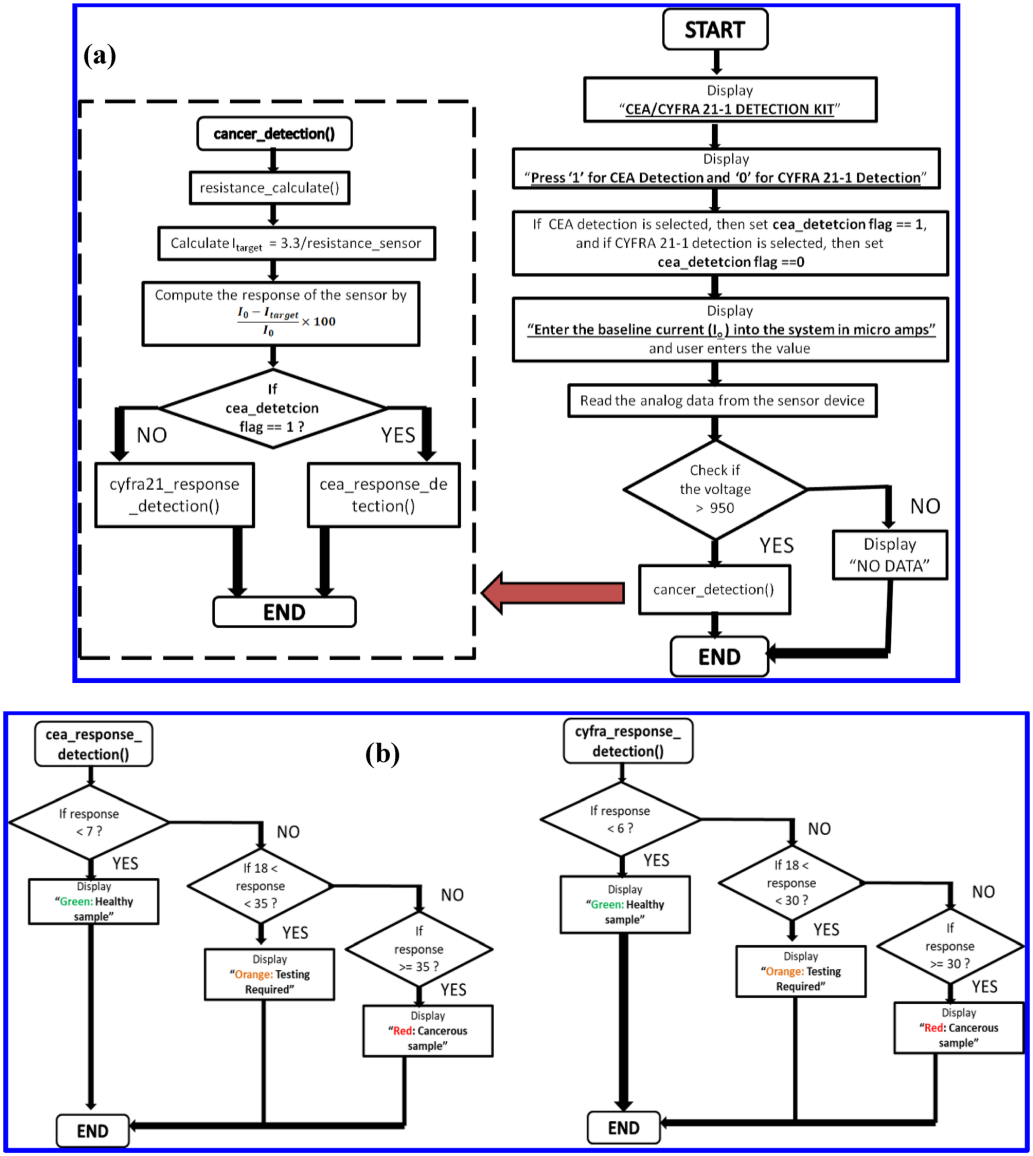
(**a**) Flowchart of the algorithm developed for the prediction of CEA/CYFRA 21-1 concentration. (**b**) Subroutines for the main flowchart.

Furthermore, the sensor responses were shown to be non-overlapping across CEA and CYFRA 21-1 concentrations (Fig. 4). This helped in developing a simple algorithm for predicting CEA and CYFRA 21-1 concentrations accurately. Figure 5 shows the flowchart of the proposed algorithm. The algorithm starts with the user choosing which antigen response (CEA/CYFRA 21-1) has to be calculated. A flag is raised high if CEA detection is chosen and it is set to low if CYFRA 21-1 is chosen. Next, the baseline current (I_0_) has to be entered when a particular sensor is plugged into the circuit. As soon as I_0_ is entered, the auto range ohmmeter calculates the resistance of the device and converts it to current by following ohms law. Next, the current flowing across the device after incubating the antigen for an hour (which is optimum incubation time, Fig. S6), was measured and recorded as I_target_. The response of the sensor device is calculated using Eq. (1) (“Biosensor characterizations”). The flag set earlier is examined and checked if the measurement is for CEA or CYFRA 21-1. Based on the value of the response, as calculated in the previous step, the program enters into one of three subroutines which are allocated as green, orange and red.

The green zone gets displayed on the LCD when the response is calculated to be less than 14% for CEA or less than16% for CYFRA 21-1 which indicates that the antigen concentrations are less than 5 ng/mL and are in the healthy range. The orange zone represents the range where the concentrations of CEA and CYFRA 21-1 are higher than the healthy range 5–20 ng/mL. It has been reported in the literature that along with the cancer patients, some of the healthy samples might also comprises CEA or CYFRA 21-1 of concentrations ~ 20 ng/mL^54^. Hence, if the responses of the sensors are found in this range, then an alert message for detailed testing may be given to the user. The red zone comprises all the responses above 35% for CEA and 30% for CYFRA 21-1 as these responses indicate the concentrations of the antigens to be higher than 20 ng/mL which can only be the case, if the patient has cancer.

#### Assembling the portable readout circuit

**Figure 6:**
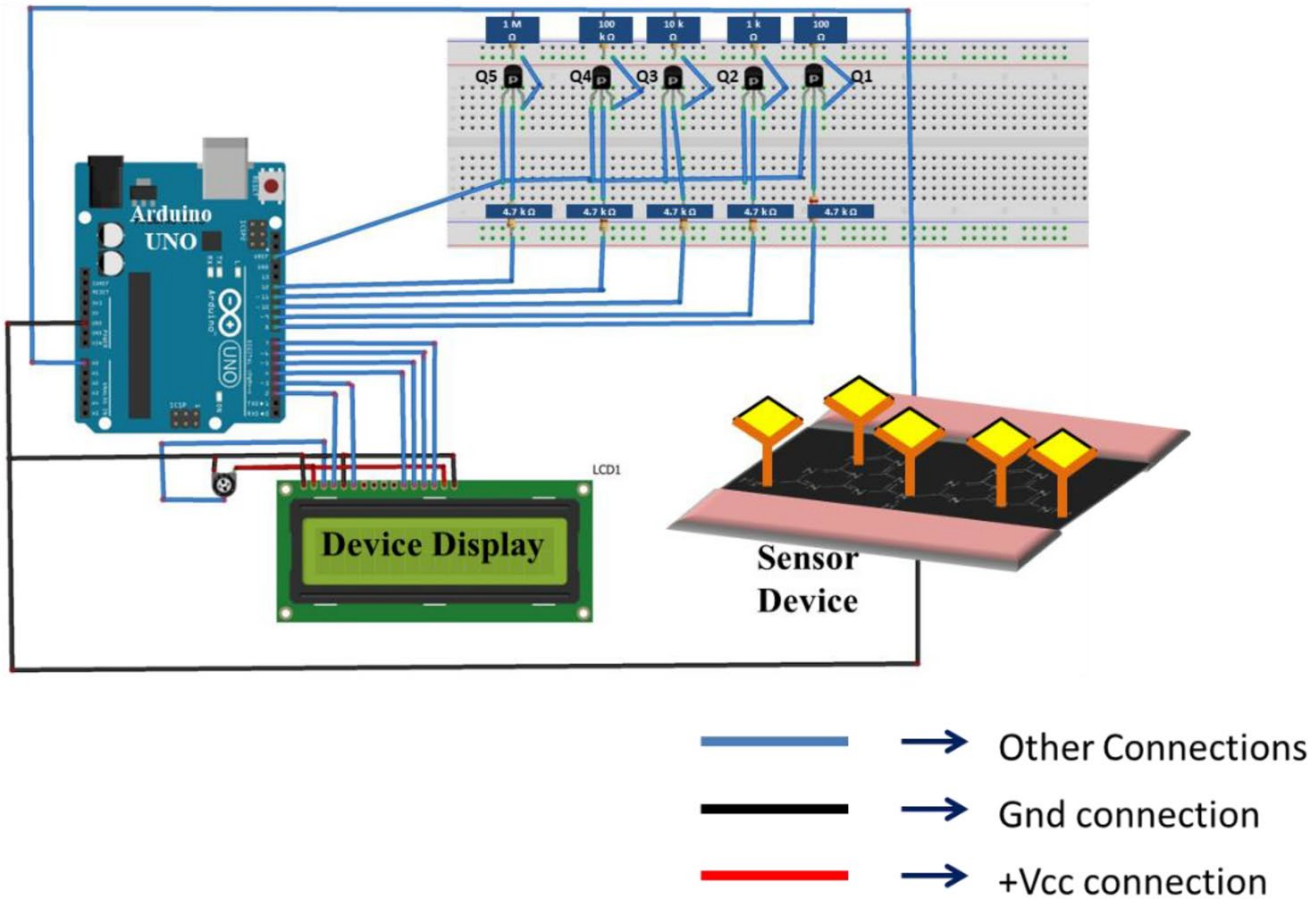
Diagram of the interfacing readout circuit.

An Arduino UNO board, an LCD, and an auto range ohmmeter were used to build the read-out circuit for the developed electronic biosensor (Fig. 6). A few challenges were posed during the assembling of the circuit. To begin with, the Arduino UNO cannot be used to directly measure the current flowing through a device. Also, the device-to-device baseline variations in of the biosensors posed the next challenge in getting a generic circuit that can work for a wide range of baseline of the sensors. As a remedy to both the problems, an auto range ohmmeter circuit was designed and tested for measuring the resistances of the biosensors and then convert the value to current using the ohms law. The steady voltage source of 3.3 V from Arduino UNO was utilized directly to facilitate the measurements. The auto-range multimeter uses five PNP transistors Q1–Q5 which are operated as electrical switches in the circuit. The UNO’s 3.3 V pin is linked to the emitter terminals of all the five transistors. The collector of each of the transistor is connected to a resistor of different values (100 Ω–1MΩ). The circuit is such that only one transistor is active at any given moment, while the rest are in cut-off. This enables the auto switching of resistance to a comparable value of the sensor device as shown in the Fig. 6.

The responses of the sensors were measured using the readout circuit and were evaluated by the algorithm loaded into the UNO board's microcontroller. This circuit rendered the devices with portability by reducing the dimensions significantly. The prototype that comprised of the sensors and the interfacing circuit was tested for different concentrations of pure CEA and CYFRA 21-1 and the error in the measurements ranged from 2 to 4%.

### Human saliva test results

The developed prototype was tested with human saliva samples of nine oral squamous cell carcinoma (OSCC) patients and five healthy (control) samples. The saliva collection protocol and the exclusion criteria followed are detailed in Supplementary Information S1. 6 μL of the thawed saliva was dropped on the developed sensors and the responses were recorded using the prototype. The response values as measured by the prototype were cross validated by measuring the currents flowing through the sensors using the DAQ.

**Figure 7:**
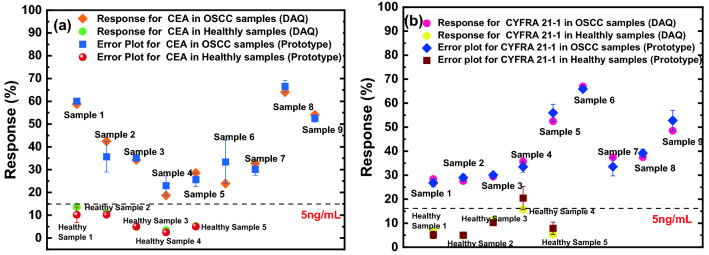
Real time saliva samples test results of (**a**) CEA (**b**) CYFRA 21-1.

It was observed that the concentrations of both CEA and CYFRA 21-1 in saliva samples of OSCC patients were more than 5 ng/mL as can be seen in Fig. 7. The concentrations of the antigens in saliva of healthy subjects however, varied from 1 pg/mL to a little less than 5 ng/mL. Hence, the threshold concentrations of both the antigens as reported by literature (5 ng/mL) were found to be followed in all the tested fourteen live samples. In order to cross-validate the performance of the developed prototype, the concentrations of CEA present in all the real-life samples were assessed using a commercially purchased ELISA Kit. The detection range of the test kit was 0.3–250 ng/mL. The comparison of the results obtained through ELISA kit and those observed through the developed sensor devices are shown in the Table 2. The raw data as obtained from the kit has been included as Fig. S7. The results show that the electronic biosensors are highly efficient in detecting CEA and CYFRA 21-1 and have high potential to be used in clinical diagnostics. In addition, the developed sensors can detect as low as 1 pg/mL concentrations of both the antigens which was not readily possible with the ELISA kit.

**Table 2:** Comparison of the CEA concentration as detected by the developed prototype and ELISA.

Sample no.	Concentration predicted by ELISA (ng/mL)	Concentration predicted by developed prototype (ng/mL)	OSCC/healthy sample
1	250	250	OSCC sample
2	102	100–400	OSCC sample
3	22.17	20–100	OSCC sample
4	6.47	5–10	OSCC sample
5	13.1	10–20	OSCC sample
6	10.685	10–20	OSCC sample
7	19.04	10–20	OSCC sample
8	150.81	150	OSCC sample
9	201.6	200	OSCC sample
10	2	1–5	Healthy sample
11	1.5	1	Healthy sample
12	0.0836	< 0.001	Healthy sample
13	0.0887	< 0.001	Healthy sample
14	0.0578	< 0.001	Healthy sample

**Figure 8:**
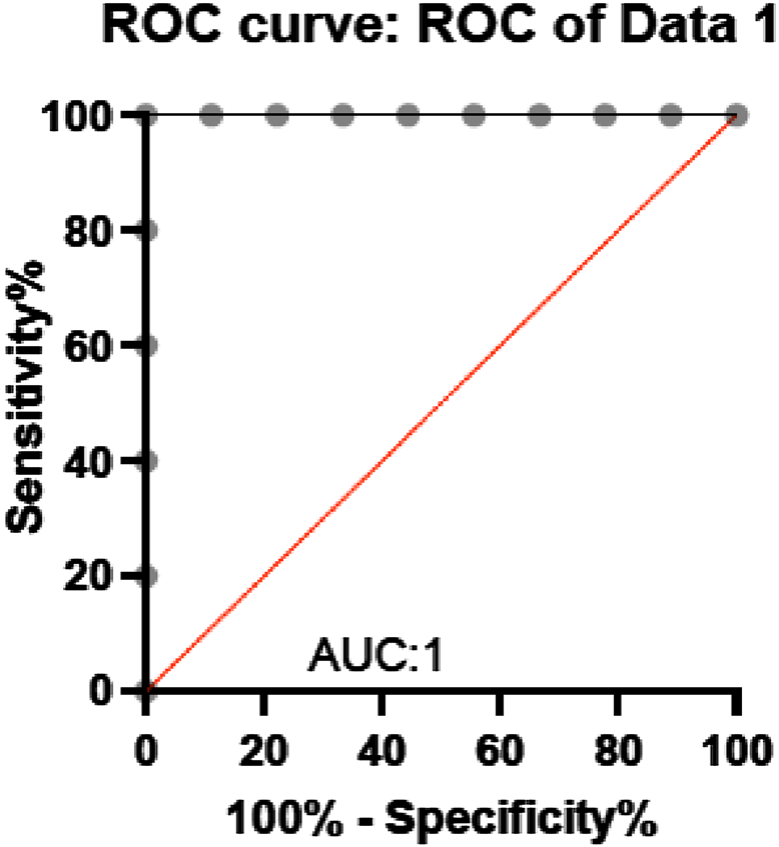
ROC data curve for CEA.

This validation of the performance of the developed prototype using ELISA kit could not be performed for CYFRA 21-1 as the kit was not readily available. In order to evaluate the diagnostics capabilities of the antigens and the specificity of the biomarkers to the OSCC cases, receiver operating characteristics (ROC) curve was plotted for CEA as we had the ELISA test results available for the same. Figure 8 shows the ROC generated using the concentration of the antigens found in OSCC samples and in normal samples. It can be seen that the area under the curve is 1 which indicates high sensitivity and specificity of CEA in discriminating between cancer cases and healthy samples.

## Discussion

This study reports a simple, label-free, rapid and effective way for detecting CEA and CYFRA 21-1 antigens by monitoring the change in current flowing through the rGO/MEL/antibody/BSA sensors. The sensors were tested at room temperature for eight different concentrations of the two cancer biomarkers ranging between 1 pg/mL and 800 ng/mL. The response of the rGO/MEL/anti-CEA/BSA devices were found to vary between 7.29 and 59.1% for 1 pg/mL to 800 ng/mL CEA and the response of rGO/MEL/anti-CYFRA/BSA ranged from 6.1 to 64% for 1 pg/mL to 800 ng/mL CYFRA 21-1. The LOD were statistically determined to be 0.148 pg/mL and 0.4 pg/mL for CEA and CYFRA 21-1 respectively. The sensors were found highly selective to the respective antigen, stable even when tested after 14 days and were highly repeatable when tested with devices with varying baseline currents. A portable read-out circuit was developed and the prediction of the antigen concentrations was aided by an algorithm that was developed based on response of the sensors for the different concentrations of CEA and CYFRA 21-1. The developed prototype was tested with 14 live samples and the predicted concentrations of CEA were found to be excellent agreement with the commercially purchased ELISA kit. It is hoped that this research will lead to the development of portable and easy-to-use POC devices for detecting the cancer biomarkers which might subsequently lead to the early diagnosis of the terminal diseases.

## Methods

### Materials

ITO coated glass sheets (resistivity of ~ 20 Ω/sqm) were procured from Shilpa Enterprises, India. Abcam provided CEA, CYFRA 21-1 antigens and monoclonal anti-IgG CEA and anti-IgG CYFRA 21-1 antibodies. Melamine, triazine, phosphate buffer saline (PBS) solution, bovine serum albumin (BSA) and all the other chemicals were purchased from Sigma Aldrich. All the experiments were carried out with ultrapure water (18.11 M Ω cm).

### Synthesis of rGO

The process began with the oxidation and subsequently, exfoliation of bulk graphite powder to GO using the modified Hummer’s method following the steps reported earlier^40^. Next, the GO was reduced chemically using ascorbic acid (AA) to get the rGO. Briefly, the obtained GO dispersion (0.1 mg/mL) was taken, 4 g AA was added to it and the solution was then set for stirring at 60 °C for 40 min on a magnetic stirrer. The hence obtained black dispersion was centrifuged for 60 min at 2000 revolutions per minute (rpm) and the precipitate was collected. The rGO sample obtained after the centrifuge was then rinsed multiple times with ethanol and de-ionized (DI) water before being dried at 120 °C for 24 h.

### Fabrication of biosensor

Commercially purchased ITO coated glasses were used as the base substrate for fabricating the biosensor device as the device needed conducting electrodes which were defined using the conducting ITO layers. To get the conductive electrodes at the two edges of the device, the ITO film of width of around 1 mm was etched out from the centre of the substrate using aqua regia. Next, the etched ITO coated glasses were washed with DI water and dried. The resistances across the two ITO strips were measured and found to be open circuited to ensure proper removal of the ITO film from the desired portion of the biosensors. The etched ITO/glasses were then coated with rGO (20 μL, 1 mg/mL) and dried at 50 °C for 30 min in ambient conditions. For the antibody immobilizing layers, MEL and triazine were dispersed in DMF to get a uniform dispersion (4 mg/mL). 6 μL of the dispersion was coated on the rGO layer and then dried at 80 °C for 5 min such that the DMF gets vaporized completely. Next, anti CEA/CYFRA 21-1 (6 μL) were drop casted onto the rGO/polymer devices and were incubated at 4 °C for an hour. After the first incubation, the samples were retrieved and washed with PBS solution to eliminate the unbounded antibodies on the surface of the biosensors. These unbound antibodies are expected to create agglomerates on the surface of the biosensors thereby, hindering the antigen–antibody bindings. Next, BSA (3 μL) was coated on the rGO/polymer/antibody to block the non-binding sites of the sensors. After that, different concentrations of (1 pg/mL to 800 ng/mL) CEA and CYFRA 21-1 (6 μL) were dropped onto the rGO/polymer/antibody devices followed by the final incubation at 4 °C for an hour. A constant 3.3 V DC was applied across the devices and the current flowing through the device after coating rGO and post antigen binding were measured.

### Material characterizations

The morphology of the sensor surface after introduction of different layers was studied using Carl Zeiss Gemini 300 FESEM. To carefully examine the amount of nitrogen atoms that in turn, validates the presence/absence of amine groups which play an important role in antigen binding, present in the MEL and triazine, Oxford EDS attached with FESEM was used. Nicolet Impact-400 FTIR spectrometer was used to record infrared spectra for solid samples.

### Electrical characterizations

#### Sensor characterizations

The change in the current flowing through the device after coated rGO and post antigen binding was taken as the signal of the developed sensors. A constant voltage was supplied to the biosensors through GPD-3303D regulated power supply (RPS). Keithley data acquisition unit (DAQ6510) was used to log the electrical current flowing through the devices. The data was continuously fed to the computer through a graphical interface provided with the DAQ.

#### Read-out circuit for portable sensor

To develop a portable sensor device, a compact interfacing read-out circuit was designed using Arduino Uno and an auto range ohm meter circuit. The need for an auto range ohm meter circuit rose because the amplitude of the current to be measured from the sensor device was tens to hundreds of μA. This was particularly challenging because very small currents (μA ranges) often require the use of special charge integration devices, which accumulate charge over a controlled time. The use of an Arduino UNO in such scenarios would be limited to communicating with the ADC. An attempt to solve this problem using a simple potential divider circuit was made but that circuit was not able to cater to a varied range of baseline currents of different devices. Therefore, moving one step ahead, an auto range ohm meter was designed which works by using voltage divider technique with different values of resistors ranging from 100 Ω to 1 MΩ connected with a pnp transistor. This circuit forms an electronic switch arrangement which switches to the desired resistance based on the resistance of the sensor device through a C program dumped onto the Arduino UNO.

### Saliva collection

The saliva samples of all the participants were collected with the informed consents of the concerned subjects. The sample collection was done after acquiring the appropriate approvals (KIMS:ETHCS COMM:516:2021-22) from the Karnataka Institute of Medical Sciences, Hubballi Ethics Committee (Reg No. ECR/486/Inst/KA/2013/RR-16). The experiments were conducted in accordance with relevant guidelines and regulations.

### ELISA protocol

The levels of CEA present in the human saliva samples were determined using a commercial 96-well plate ELISA kit (no. ab99992; Abcam). The kit had biotinylated human CEA antibodies inoculated into the wells. 100 μL of standard and saliva samples were pipetted into the wells to facilitate the binding of immobilized antibodies to the CEA present in the saliva samples. The plate was covered and kept for 2.5 h incubation at room temperature with gentle shaking. After incubation, the next step was to discard the solution and clean the plates with wash buffer. After cleaning, 100 μL of 1X biotinylated CEA detection antibody was added to each well and incubated for 1 h at room temperature with gentle shaking. Next, 100 μL of 1X Horseradish peroxidase-conjugated (HRP) streptavidin was pipetted into the wells after washing the unbound biotinylated antibodies using wash buffer and then 100 μL of tetramethylbenzidine (TMB) substrate solution was added. The yellow color was observed after following the above steps. Lastly, 50 μL of stop solution was added to the wells and a change in the color from yellow to blue was observed, the contrast of which was in proportion to the concentrations of bound CEA. The absorbance value was calculated at 450 nm and the concentrations of CEA in saliva samples were determined using the standard curves.

## Supplementary Information

Supplementary Information.

## Data Availability

All data generated or analysed during this study are included in this published article (and its supplementary information files).

## References

[1] Rana JS, Khan SS, Lloyd-Jones DM, Sidney S (2021). Changes in mortality in top 10 causes of death from 2011 to 2018. J. Gen. Intern. Med..

[2] Asia S, Asia S, Hdi H (2020). All cancers..

[3] Mathur, P., Sathishkumar, K., Chaturvedi, M. & Das, P. Cancer Statistics , 2020 : Report From National Cancer Registry Programme , India abstract. 1063–1075 (2021). 10.1200/GO.20.00122. 10.1200/GO.20.00122 PMC7392737 32673076

[4] National Cancer Institute. Statistics at a Glance: The Burden of Cancer in the United States. *Cancer Stat.* OMB No.: 0925–0642 (2017).

[5] Holtedahl K (2020). Challenges in early diagnosis of cancer: The fast track. Scand. J. Prim. Health Care.

[6] Walter FM (2015). Symptoms and other factors associated with time to diagnosis and stage of lung cancer: A prospective cohort study. Br. J. Cancer.

[7] Berrington De González, A. *et al.* Projected cancer risks from computed tomographic scans performed in the United States in 2007. *Arch. Intern. Med.* **169**, 2071–2077 (2009). 10.1001/archinternmed.2009.440 PMC6276814 20008689

[8] Bradley SH (2019). Sensitivity of chest X-ray for detecting lung cancer in people presenting with symptoms: A systematic review. Br. J. Gen. Pract..

[9] Petty TL (2000). The early identification of lung carcinoma by sputum cytology. Cancer.

[10] Coley SM, Crapanzano JP, Saqi A (2015). FNA, core biopsy, or both for the diagnosis of lung carcinoma: Obtaining sufficient tissue for a specific diagnosis and molecular testing. Cancer Cytopathol..

[11] Prabhakar B, Shende P, Augustine S (2018). Current trends and emerging diagnostic techniques for lung cancer. Biomed. Pharmacother..

[12] Syedmoradi, L., Norton, M. L. & Omidfar, K. Point-of-care cancer diagnostic devices: From academic research to clinical translation. *Talanta* **225**, (2021). 10.1016/j.talanta.2020.122002 33592810

[13] Arya SK, Bhansali S (2011). Lung cancer and its early detection using biomarker-based biosensors. Chem. Rev..

[14] Zamay TN (2017). Current and prospective protein biomarkers of lung cancer. Cancers (Basel)..

[15] Radhika T, Jeddy N, Nithya S, Muthumeenakshi RM (2016). Salivary biomarkers in oral squamous cell carcinoma—An insight. J. Oral Biol. Craniofacial Res..

[16] Rubins JB, Dunitz J, Rubins HB, Maddaus MA, Niewoehner DE (1998). Serum carcinoembryonic antigen as an adjunct to preoperative staging of lung cancer. J. Thorac. Cardiovasc. Surg..

[17] Nakata B, Takashima T, Ogawa Y, Ishikawa T, Hirakawa K (2004). Serum CYFRA 21–1 (cytokeratin-19 fragments) is a useful tumour marker for detecting disease relapse and assessing treatment efficacy in breast cancer. Br. J. Cancer.

[18] Al-Shagahin H, Alkotyfan K, Müller HH, Sesterhenn AM, Werner JA (2009). Cyfra 21–1 as a serum tumor marker for follow-up of patients with laryngeal and hypopharyngeal squamous cell carcinoma. Anticancer Res..

[19] Manjunath, D. *et al.* Detection and evaluation of ELISA analysis for the circulating cancers sera antigens by monoclonal antibody UNIVmAB and pembrolizumAB. **4**, (2020).

[20] Rai GP, Venkateswaran KS (1992). Limitations and practical problems in enzyme-linked immunosorbent assays. Def. Sci. J..

[21] Qi H, Zhang C (2020). Electrogenerated chemiluminescence biosensing. Anal. Chem..

[22] Wang H, Wang X, Wang J, Fu W, Yao C (2016). A SPR biosensor based on signal amplification using antibody-QD conjugates for quantitative determination of multiple tumor markers. Sci. Rep..

[23] Wang H (2016). Photoelectrochemical immunosensor for detection of carcinoembryonic antigen based on 2D TiO2 nanosheets and carboxylated graphitic carbon nitride. Sci. Rep..

[24] Kumar S (2019). Electrochemical paper based cancer biosensor using iron oxide nanoparticles decorated PEDOT:PSS. Anal. Chim. Acta.

[25] Xiao L (2017). Colorimetric biosensor for detection of cancer biomarker by Au nanoparticle-decorated Bi2Se3 nanosheets. ACS Appl. Mater. Interfaces.

[26] Sandbhor Gaikwad, P. & Banerjee, R. Advances in point-of-care diagnostic devices in cancers. *Analyst* **143**, 1326–1348 (2018). 10.1039/c7an01771e 29469148

[27] Crivianu-Gaita V, Thompson M (2016). Aptamers, antibody scFv, and antibody Fab’ fragments: An overview and comparison of three of the most versatile biosensor biorecognition elements. Biosens. Bioelectron..

[28] Webster, D. M., Henry, A. H. & Rees, A. R. Antibody-antigen interactions. (1994).

[29] Nguyen HH, Lee SH, Lee UJ, Fermin CD, Kim M (2019). Immobilized enzymes in biosensor applications. Materials (Basel)..

[30] Rashid JIA, Yusof NA (2017). The strategies of DNA immobilization and hybridization detection mechanism in the construction of electrochemical DNA sensor: A review. Sens. Bio-Sensing Res..

[31] Putzbach W, Ronkainen NJ (2013). Immobilization techniques in the fabrication of nanomaterial-based electrochemical biosensors: A review. Sensors (Switzerland).

[32] Chiu ML, Goulet DR, Teplyakov A, Gilliland GL (2019). Antibody structure and function: The basis for engineering therapeutics. Antibodies.

[33] Gao, S., Guisán, J. M. & Rocha-Martin, J. Oriented immobilization of antibodies onto sensing platforms—A critical review. *Anal. Chim. Acta* **1189**, (2022). 10.1016/j.aca.2021.338907 34815045

[34] Zhou, J., Li, S., Noroozifar, M. & Kerman, K. Graphene oxide nanoribbons in chitosan for simultaneous electrochemical detection of guanine, adenine, thymine and cytosine. *Biosensors* **10**, (2020). 10.3390/bios10040030 PMC7236021 32230779

[35] Özcan B, Sezgintürk MK (2016). Graphene oxide based electrochemical label free immunosensor for rapid and highly sensitive determination of tumor marker HSP70. Talanta.

[36] Tolleson, W., Diachenko, G. & Heller, D. Background paper on the chemistry of melamine alone and in combination with related compounds. *WHO Expert Meet. Toxicol. Heal. Asp. Melamine Cyanuric Acid* 18 (2009).

[37] Shen M, Rusling JF, Dixit CK (2017). Site-selective orientated immobilization of antibodies and conjugates for immunodiagnostics development. Methods.

[38] Rowley-Neale, S. J., Randviir, E. P., Abo Dena, A. S. & Banks, C. E. An overview of recent applications of reduced graphene oxide as a basis of electroanalytical sensing platforms. *Appl. Mater. Today* **10**, 218–226 (2018).

[39] Han J, Ma J, Ma Z (2013). One-step synthesis of graphene oxide-thionine-Au nanocomposites and its application for electrochemical immunosensing. Biosens. Bioelectron..

[40] Joshi, S., Guruprasad, G., Kulkarni, S. & Ghosh, R. Reduced graphene oxide based electronic sensors for rapid and label-free detection of CEA and CYFRA 21-1. *IEEE Sens. J.* **XX**, 1–1 (2021).

[41] Peng, H. P., Lee, K. H., Jian, J. W. & Yang, A. S. Origins of specificity and affinity in antibody-protein interactions. *Proc. Natl. Acad. Sci. U. S. A.* **111**, (2014). 10.1073/pnas.1401131111 PMC4084487 24938786

[42] Wang J (2018). Increased CYFRA 21–1, CEA and NSE are prognostic of poor outcome for locally advanced squamous cell carcinoma in lung: A Nomogram and recursive partitioning risk stratification analysis. Transl. Oncol..

[43] Pachauri N, Dave K, Dinda A, Solanki PR (2018). Cubic CeO2 implanted reduced graphene oxide-based highly sensitive biosensor for non-invasive oral cancer biomarker detection. J. Mater. Chem. B.

[44] Wang H, Rong Q, Ma Z (2016). Polyhydroquinone-graphene composite as new redox species for sensitive electrochemical detection of cytokeratins antigen 21–1. Sci. Rep..

[45] Augustine S, Kumar P, Malhotra BD (2019). Amine-functionalized MoO3@RGO nanohybrid-based biosensor for breast cancer detection. ACS Appl. Bio Mater..

[46] Lai Y (2019). A sandwich-type electrochemical immunosensor using polythionine/AuNPs nanocomposites as label for ultrasensitive detection of carcinoembryonic antigen. Mater. Express.

[47] Yuan X (2016). Combinatorial vibration-mode assignment for the FTIR spectrum of crystalline melamine: A strategic approach toward theoretical IR vibrational calculations of triazine-based compounds. J. Phys. Chem. A.

[48] Castelar S (2013). Supramolecular dendrimers based on the self-assembly of carbazole-derived dendrons and triazine rings: Liquid crystal, photophysical and electrochemical properties. J. Mater. Chem. C.

[49] Ruid, M., Miguel, A. Á., Cruz-quesada, G., Rivera-utrilla, J. & Manuel, S. Ethylparaben degradation. (2020).

[50] Cruz M (2017). Bare TiO 2 and graphene oxide TiO 2 photocatalysts on the degradation of selected pesticides and influence of the water matrix. Appl. Surf. Sci..

[51] Du FP (2018). PEDOT:PSS/graphene quantum dots films with enhanced thermoelectric properties via strong interfacial interaction and phase separation. Sci. Rep..

[52] Saha B, Songe P, Evers TH, Prins MWJ (2017). The influence of covalent immobilization conditions on antibody accessibility on nanoparticles. Analyst.

[53] Jozghorbani M, Fathi M, Kazemi SH, Alinejadian N (2021). Determination of carcinoembryonic antigen as a tumor marker using a novel graphene-based label-free electrochemical immunosensor. Anal. Biochem..

[54] Honarmand M, Farhad-Mollashahi L, Nakhaee A, Nehi M (2016). Salivary levels of ErbB2 and CEA in oral squamous cell carcinoma patients. Asian Pac. J. Cancer Prev..

