# Supplementary material for: Simple diagnosis of cancer by detecting CEA and CYFRA 21-1 in saliva using electronic sensors
Source: Sci Rep. 2022 Sep 12;12:15315. doi: 10.1038/s41598-022-19593-8 (PMC9468134; doi:10.1038/s41598-022-19593-8)
Supplement: Supplementary file 1 — Supplementary Information. [file 41598_2022_19593_MOESM1_ESM.docx]

**Supplementary Information**

**Simple Diagnosis of Cancer by Detecting CEA and CYFRA 21-1 in Saliva Using Electronic Sensors**

Sowmya Joshi^1^, Shashidhar Kallappa^2^, Pranjal Kumar^3^, Sudhanshu Shukla^3^, Ruma Ghosh^1^*

^1^Department of Electrical Engineering, Indian Institute of Technology Dharwad, Karnataka – 580011, India

^2^Department of Surgical Oncology, Karnataka Institute of Medical Sciences, Hubli, Karnataka–580029, India

^3^Department of Biosciences and Bioengineering, Indian Institute of Technology Dharwad, Karnataka – 580011, India

^*^Email: [rumaghosh@iitdh.ac.in](mailto:rumaghosh@iitdh.ac.in)


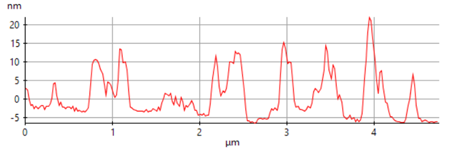


**nm**

**10**


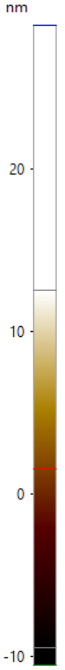


**-10**

**0**

**20**


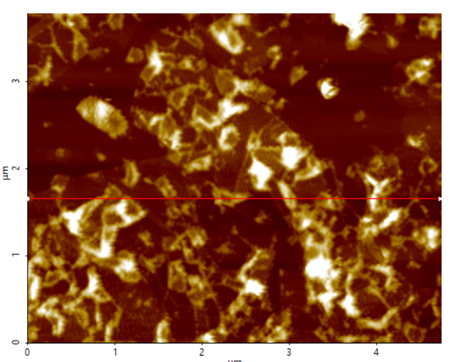


**4**

**3**

**2**

**1**

**0**

**4**

**3**

**2**

**1**

**0**

**µm**

**µm**

**Fig. S1** AFM image of the GO


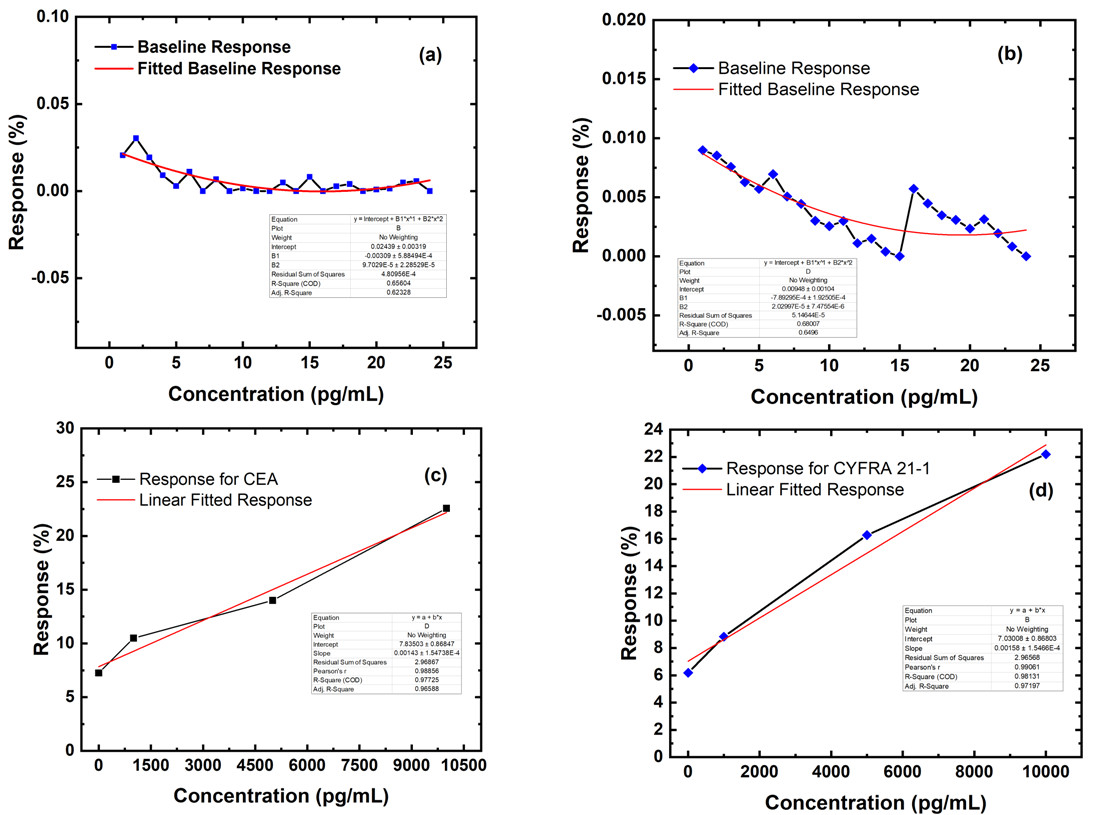


**Fig. S2 (a)** Response and linear fitted response of the sensor to the blank measurements for CEA (baseline current) **(b)** Response and linear fitted response of the sensor to the blank measurements for CYFRA 21-1 (baseline current) **(c)** Linear-fitted response for CEA **(d)** Linear-fitted response of the sensors for CYFRA 21-1

In order to calculate the LOD, it was necessary to ascertain the slope from a linear fitted response Vs concentrations graph. The fitting was observed to be non-linear if we considered the whole range of the concentrations of CEA and CYFRA 21-1 for which the sensors were tested. Hence, the linear fitting of the response Vs concentrations was done considering 1pg/mL to 10ng/mL of CEA and CYFRA 21-1.

**Fig. S3** Response of rGO/MEL/antibody/BSA devices fabricated by varying the concentrations of MEL to 20 ng/mL of CEA and CYFRA 21-1


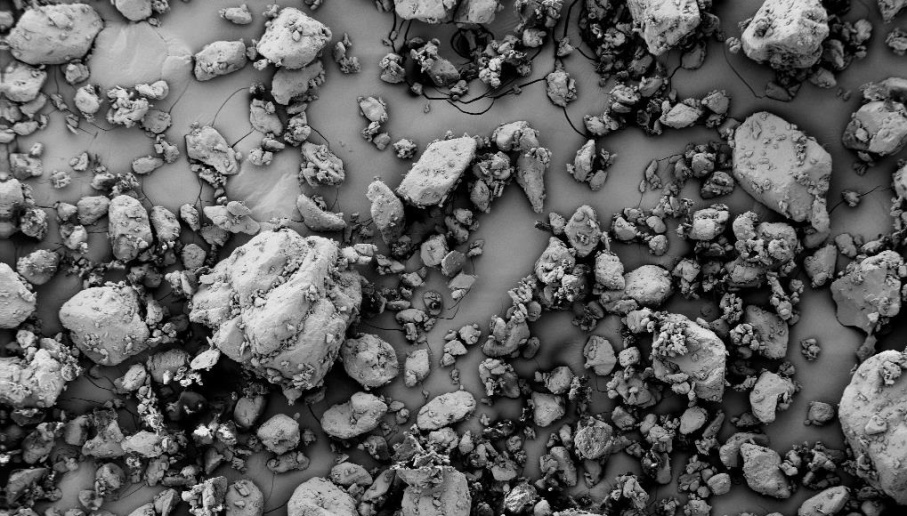


**100 µm**

**Fig. S4** FESEM image of 8 mg/mL MEL sample dispersed in DMF


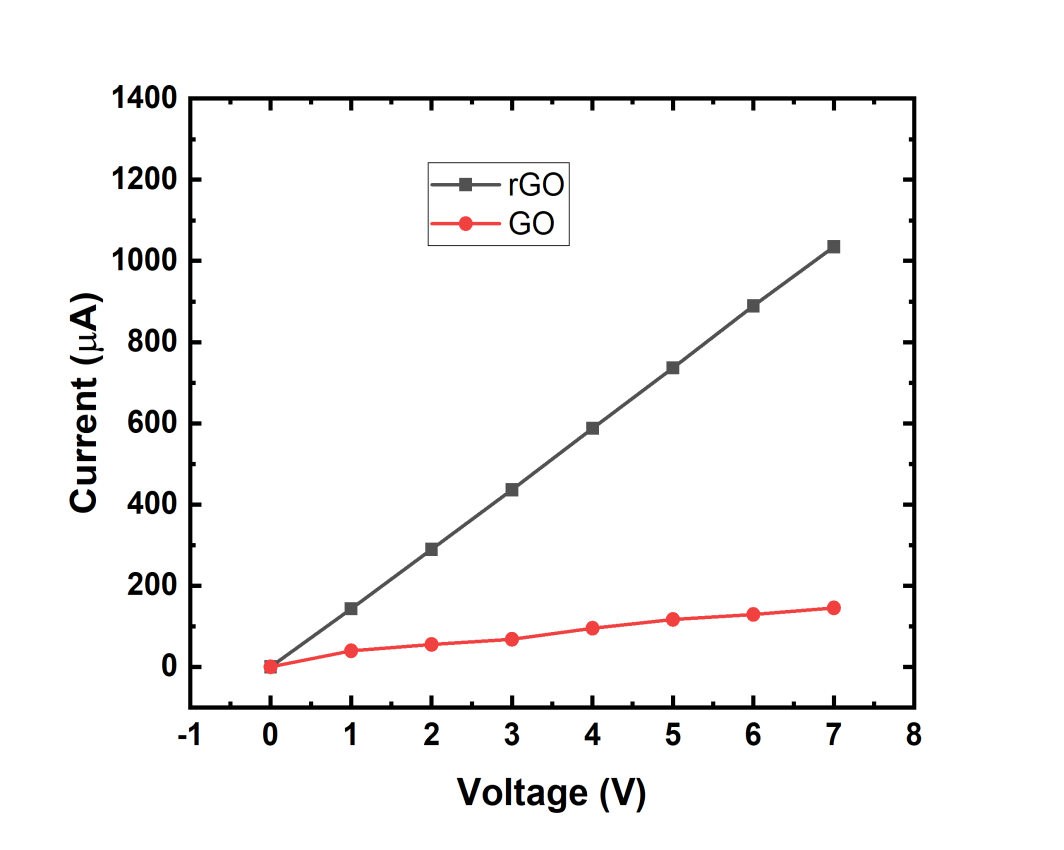


**Fig. S5** I-V characteristics of GO AND rGO


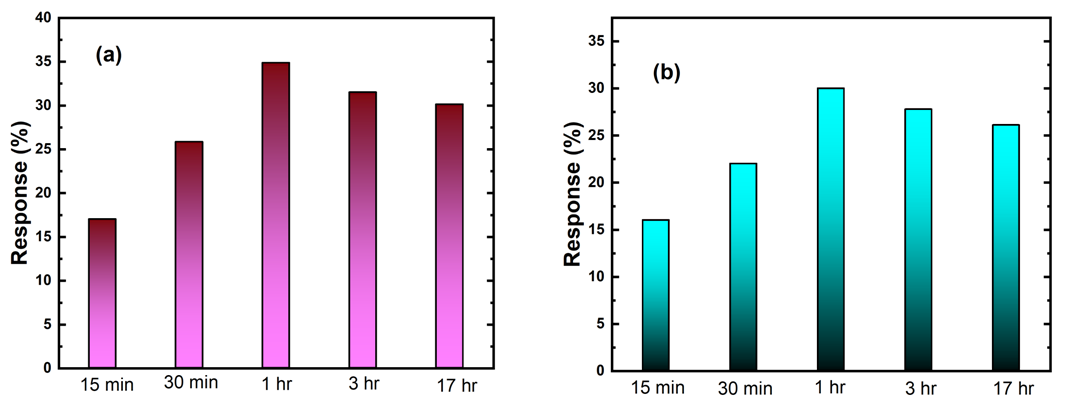


**Fig. S6** Incubation Time Plots (a) CEA (b) CYFRA-21-1

The antigens were incubated on the sensors at 4 °C for one hour. It was necessary to test the stability of the fabricated sensors. So, one device was prepared by incubating 20ng/mL of CEA antigen on rGO/MEL/anti-CEA sensor and 20ng/mL CYFRA 21-1 antigen on rGO/MEL/anti-CYFRA 21-1 sensor. The devices were incubated for 17 hours and the current flowing the device was measured after each hour. Figure S6 shows the results obtained for CEA and CYFRA -21-1 by incubating the antigen for different durations. The results (as shown in Figure S6) indicate that there is no significant amount of current change even after 17 hours of incubation starting from 1 hour of incubation time. Hence, for our measurements, we incubated the target biomarkers for 1 hour.

**Protocol followed for collecting the saliva samples**

The real-life samples tested in this work were collected at KIMS, Hubli. The samples were collected in the morning, at least two hours after eating. All the subjects were asked to wash their mouths thoroughly with water before collecting the saliva samples through passive drooling. Around 1–1.5 mL of saliva samples was collected from each subject and all the samples were stored at –80 °C immediately after collection along with protease inhibitor cocktail (1:1000 i.e., 1 μL of inhibitor was added to 1 mL saliva). This was done to preserve the proteins present in the saliva sample from getting digested. The saliva samples were thawed and centrifuged to remove insoluble material before testing them using the developed prototype and the commercially purchased ELISA Kit.

**Availability of Data and Materials**

Fig. S7 shows the data generated by the ELISA test for CEA during the study. In the 96 well plate, A-H 1 was used for creating a standard sample concentration. A-E 2 and A-E 3 were reserved for the Healthy samples (Sample 1– Sample 4). A4- A12 was reserved for testing the OSCC sample (Sample 1- Sample 9) and a replica was maintained in B4-B12.

**Fig. S7** ELSIA kit Raw Data file for CEA detection

**Exclusion criteria:**

1. The subjects suffering from long term diseases like high blood pressure, diabetes etc.

2. The subjects having any acute disease other than OSCC.

3. For healthy samples, subjects having any medical condition.
